# Responding to Emerging Diseases Requires Multi-disciplinary and One Health Training, Egypt

**DOI:** 10.29024/aogh.2372

**Published:** 2018-11-05

**Authors:** Amira Roess, Sally Lahm, Ibrahim Kabbash, Amal Saad-Hussein, Ashraf Shaalan, Ossama Rasslan, Mohamed Mohamed

**Affiliations:** 1 The George Washington University, Milken Institute School of Public Health, Department of Global Health, US; 2 State University of New York at Buffalo, Department of Epidemiology and Environmental Health Buffalo, New York, US; 3 Tanta University, Faculty of Medicine, EG; 4 National Research Centre, Environmental and Occupational Medicine Department, Giza, EG; 5 National Research Centre, Biological Anthropology Department, Giza, EG; 6 Ain Shams University, Infectious Diseases Research and Infection Control Unit, Cairo, Egypt and Scientific Council of Egyptian Fellowship Board for Infection Control, Cairo, EG; 7 The George Washington University, School of Medicine and Health Sciences, Department of Pediatrics, US

## Abstract

**Background:**

In Egypt, several infectious diseases of zoonotic origin have emerged in recent years like H1N1, MERSCoV and H5N1, the latter now endemic. Responding to these diseases requires a workforce trained in multi-disciplinary approaches to zoonotic disease research and control. It is difficult to deliver multidisciplinary and one health training globally because of the limited number of higher education programs that support such training. In low and middle-income countries where the impacts of emerging zoonotic diseases are felt more directly there is enthusiasm for such training and the use of e-technology can foster international, long-term collaborations.

**Objectives:**

To provide health training for infectious diseases research and to foster multidisciplinary collaboration.

**Methods:**

We designed and simultaneously held two training workshops, one focused on pediatric infectious diseases and another on emerging infectious diseases to meet the objective. Both workshops had pre- and post-workshop activities for multi-disciplinary methods with an emphasis on the use of mobile technologies to enhance emerging infectious diseases surveillance and research for public health professionals in Egypt. Faculty and scientists from all universities in Egypt and from the National Research Center were invited to participate.

**Results:**

85 participants attended, 31 abstracts were submitted, and over a 3 year period 3 international grant applications were submitted, and 4 abstracts were presented at international conferences. An online forum was developed to continue building collaboration.

**Conclusions:**

Interactive on-site workshops are suitable for providing multi-disciplinary training for disease surveillance, research and disease control. Participants shared the opinion that grant proposal and scientific manuscript writing were important skills that they felt they did not have. Long term investments in workshops of this nature are needed to build upon the excitement generated by these activities.

## Introduction

“One Health” aims to approach zoonotic infectious diseases from a complex systems ideology and an ecological studies lens, including the interactions between risk factors at the environmental, individual, and social levels which all influence the emergence and spread of disease [1]. There is a significant need for multi-disciplinary and One Health teams of public health researchers, veterinarians, wildlife specialists, clinicians, environmental health specialists and others to collaborate on emerging zoonotic infectious diseases (EZIDs) research and surveillance [2]. In Egypt, several infectious diseases of zoonotic origin have emerged in recent years, including Hepatitis E Virus, MERSCoV and H5N1, the latter now endemic [3,4,5]. These diseases disproportionately affect poorer populations in regular contact with wildlife and livestock, for example, H5N1 and HEV have been shown to disproportionately affect vulnerable groups such as those who take care of livestock (who also are usually poor), those who do not have access to adequate sanitation and clean water, and children and pregnant women [4,6]. In most countries a culture of multi-disciplinary education does not exist within which to build One Health training programs and low and middle income countries (LMICs) are most affected by this dearth in training. Finally, the use of e-technology, especially mobile phones, to enhance surveillance and public health research and interventions continues to show promise (although with limited effectiveness data to date) [7].

While online training programs provide theoretical knowledge and are conveyed as solutions to meet the demand for training [8], they are still inadequate in providing practical multi-disciplinary or One Health training [9]. Thus, the traditional training model favored by Western donors which involves a single on-site workshop focused on one scientific area with limited follow-up must be expanded to include follow-up on-site workshops or training sessions enhanced with web based activities, and must incorporate multi-disciplinary and One Health scientists.

Our aim was to provide training on multi-disciplinary research methods to address emerging, zoonotic and infectious diseases with an emphasis on the use of mobile technology led by a binational One Health team. In order to foster collaboration between disciplines, we simultaneously held a pediatric infectious disease workshop that overlapped with the zoonotic diseases workshop. We also wanted to build a network of scientists interested in building long term collaborations.

## Methods/Activities

We developed and simultaneously held two training workshops, one focused on pediatric infectious diseases and another on emerging infectious diseases. Both workshops offered pre- and post-workshop activities to a small set of trainees. Similar approaches have been used for several workshops in LMICs [9]. In April 2013, faculty from George Washington University and the Egyptian National Research Centre (NRC), held these two 4-day workshops at NRC’s headquarters in Cairo. The interactive workshops included small group exercises that integrated pediatric infectious disease specialists and scientists working in the One Health area. These activities were conducted during each day of the joint workshop and during brainstorming sessions to identify priority research and practice topics.

## Setting

The majority of emerging infectious diseases that affect humans are of zoonotic origin; more than 75% of emerging zoonoses are of wildlife origin. Yet few physicians recognize this, and consequently there is a lack of professional activities that bring together human, animal, and environmental health.

## Training activities

We designed workshops for public health researchers and practitioners, physicians, veterinarians, biologists, ecologists, environmental sciences, and infectious diseases specialists. Participants were eligible to attend if they were fluent in English, had at least a master’s degree and were engaged in infectious diseases research or practice. The workshops were advertised through a website and by participating organizations and regional networks. Registration was open to all scientists in Egypt. Trainees were selected on the basis of submitting an abstract, a curriculum vitae and a recommendation. Eighty-five participants working in hospitals or research institutes throughout Egypt attended the workshops.

## Distance pre-workshop activities

Two months prior to the workshops, we set up a conference website and released a call for proposals to solicit 350-word structured research abstracts that presented results from research activities, surveillance data or pilot data that justifies future research. This was emailed to universities and scientific research organizations. NRC and US investigators also emailed the announcement to their professional networks, paying extra attention to distribute the announcement to universities and institutions located in Upper and Lower Egypt. Over 40 abstracts were submitted and 31 were accepted and placed in 2 tracks: zoonotic (16) and neonatal/infant (15) infectious diseases. Comments were sent to authors to help improve abstracts; commonly authors had not followed the predetermined structure and/or the methods did not match the study design described. Authors were paired with one of 5 US researchers to revise their abstracts. All revised abstracts were included in the workshop booklets. Trainees were asked to identify topics of interest for future research.

## Workshop activities

Two 4-day workshops were simultaneously held with daily combined sessions. Combined sessions provided participants with an overview of: 1) the use of mobile health technology for surveillance and public health practice (Day 1); 2) One Health in action (Day 2); 3) common study designs for health research (Day 3); and 4) preparing research grants (Day 4). GWU and NRC faculty led the sessions. The majority of scientists that had an abstract accepted presented posters, and the five best abstracts in each track gave oral presentations. After the combined sessions, participants joined either the zoonotic or the infant/neonatal infectious diseases workshop for training activities. On Day 1, participants formed small teams of 4–7 participants to identify knowledge gaps in the current state of EZIDs or neonatal/infant infectious diseases in Egypt. At the end of the day, teams presented their findings. On Day 2, teams that were formed on Day 1 were paired to encourage interaction and they developed research questions that addressed gaps in knowledge identified in the previous day. On Day 3, participants matched study designs to their questions and on Day 4, participants outlined a plan to complete research proposals. The workshop leaders rotated between groups to facilitate conversations and to provide guidance on study designs and methods. At the end of each day, teams presented their work. The workshops also held combined lunch and dinners each day to further facilitate interaction between participants.

At the end of the workshops, a summary of the activities and findings of each team were compiled and distributed one week later.

A survey was conducted at the end of the workshop to identify areas for future training workshops. Participants were primarily interested in receiving further training in research methods and scientific writing.

## Post-workshop activities

During the year following the course, discussions between the participants and some of the workshop leaders continued. Three international grants were written in the year following the workshop and 4 abstracts were presented at international scientific meetings (3 posters, 1 oral). One research study to assess the burden of febrile zoonotic diseases in local hospitals was funded and is currently underway. Follow-up on-site training activities were planned and later terminated due to security concerns of the funders.

The sudden loss of funding slowed down the progress, since investigators had to devise other activities to support the network that was created. Post-workshop activities, including ongoing training and mentoring are critical for sustained impacts. Support for such activities prior to and after on-site training programs should be prioritized by donors in order to build sustainable EZID and One Health capacity. Mentoring is an important part of research training and sustained programs that facilitate mentorship are needed [10]. **Box 1** summarizes the main lessons learnt during this workshop.

**Box 1: Summary of main lessons learnt**

- The distance and on-site training was suitable for multi-disciplinary one health training and facilitated debates and discussion
- The considerable amount of pre-workshop activities was necessary to engage participants prior to the workshops
- Combining workshops allowed groups of researchers that otherwise would not have had the opportunity to meet to collaborate
- Long-term mentorship and funding is needed to create a strong network of emerging zoonotic disease experts

## Costs and human resources

The budget for this course was 2500 USD in direct costs per trainee. This covered creating and maintaining the website, managing the online forum, supporting a part-time coordinator in Egypt and one in the US, travel to Cairo for 5 experts from the US, and local expenses for trainees and local experts. The participants paid no registration fees. The interactions between Egyptian and American scientists from multiple disciplines allowed a unique opportunity for information exchange and collaboration. Participants learned a great deal from each other about the global state of EZID surveillance, programs and research as well as the use of mobiles to facilitate this work.
